# Case report: Multidrug resistant gestational trophoblastic neoplasia: focus on failure of immunotherapy and success of high-dose chemotherapy

**DOI:** 10.3389/fonc.2024.1391408

**Published:** 2024-05-13

**Authors:** Anne Enuset, Lionel Duck, Claudia Petre, Jean-Pascal Machiels, Frédéric Goffin

**Affiliations:** ^1^ Department of Gynecology and Obstetrics, Université Catholique de Louvain, Brussels, Belgium; ^2^ Onco-Hematology and Palliative Care, Clinique Saint-Pierre Ottignies, Ottignies-Louvain-la-Neuve, Belgium; ^3^ Department of Gynecology and Obstetrics, Clinique Saint-Pierre Ottignies, Ottignies-Louvain-la-Neuve, Belgium; ^4^ Institut Roi Albert II, Service d’Oncologie Médicale, Cliniques Universitaires Saint-Luc and Institut de Recherche Clinique et Expérimentale (IREC, Pole MIRO), Université Catholique de Louvain, Brussels, Belgium; ^5^ Belgian Gestational Trophoblastic Disease Reference Centre, University of Liège, Liège, Belgium

**Keywords:** gestational trophoblastic neoplasia, gynaecological neoplasia, immunotherapy, chemotherapy intensification, high-dose chemotherapy (HDT)

## Abstract

Gestational trophoblastic neoplasia (GTN) is extremely rare, but has a very good prognosis, with a cure rate close to 100%, for low-risk diseases. This article describes the case of a healthy 28-year-old nulliparous patient with GTN resistant to multiple lines of treatment. The era of immunotherapy is revolutionizing oncology, having already proved its worth in the treatment of many cancers. This article will have a specific focus on the emerging role of immunotherapy in the treatment of GTN. Unfortunately, the use of an immune checkpoint inhibitor (ICI) failed in our case, emphasizing on the necessity to clearly define the future role of immune therapy in GTN. Finally, given the rapid progression of the disease after hysterectomy, induction with Paclitaxel- Ifosfamide and then intensification with high-dose Carboplatin and Etoposide with peripheral blood stem cell support was given as a rescue therapy with still curative intent.

## 1 Introduction

GTN is a spectrum comprising invasive mole, choriocarcinoma, placental site trophoblastic tumor (PSTT) and epithelioid trophoblastic tumor (ETT), and is one of the rarest gynecological tumors. 60% of GTNs result from mole (15–20% from complete hydatidiform mole (CHM) and 0,5% from partial hydatidiform mole (PHM)), and the incidence of choriocarcinoma is only 1/40000 pregnancies (1, 2). However, their prognosis is generally excellent thanks to their high chemosensitivity, and the overall cure rate exceeds 90% (1–3).

The choice of initial treatment is based on the International Federation of Gynecology and Obstetrics (FIGO) 2000 prognostic score (4–7). In Europe, monochemotherapy is the first-line choice for low-risk GTN, but resistance occurs in 25–30% of cases (8, 9). In the event off treatment failure, another single cytostatic agent or multidrug therapy may be proposed, depending on the patient’s risk profile (10, 11). Catch-up rates with Dactinomycin and EMACO are 75% and 87% respectively. Recently, some authors have suggested that immunotherapy could be a new treatment alternative for resistant disease, and the use of Avelumab has enabled in 53% of patients to be cured (12–31).

In this paper, we will discuss the current known lines of treatment that the patient unfortunately received without achieving complete remission, with an emphasis on immunotherapy.

## 2 Case report

In 2021, a 28-year-old G1P0 woman had been treated by curettage for CHM with an initial human chorionic gonadotropin (hCG) level of 220.000 IU/L. The case was registered at the Belgian National Reference Center and pathological slides were re-examined by experts. The decrease in the hCG level was regularly monitored until it reached a negative value 3 months later. Thereafter, hCG levels remained negative for 6 months.

In January 2022, the patient consulted for abnormal vaginal bleeding. At this time, the hCG level was 37.3 IU/L (nl < 5 IU/L). Close monitoring was initiated. An ectopic pregnancy was refuted after laparoscopy and intraoperative diagnostic hysteroscopy.

In agreement with the Belgian Gestational Trophoblastic Registry and Reference Center (University Hospital of Liege), we considered the development of post-molar GTN. A thoraco-abdominal CT-scan was performed to assess the extent of the disease, which proved negative. At this stage, we assigned a score of 4 and the patient received chemotherapy with methotrexate at a dose of 1mg/kg intramuscularly on days 1, 3, 5 and 7, alternating with oral folinic acid 15 mg on days 2, 4, 6 and 8 in accordance with European recommendations. Full staging was not performed, given the low-risk situation at the time. Unfortunately, resistance to treatment was observed during the 3rd cycle.

The patient underwent an imaging work-up before each new line of treatment to exclude disease extension. The extension work-up, including pelvic and brain Magnetic Resonance Imaging (MRI) and Fluorodeoxyglucose (FDG) Positron Emission Tomography (PET) scan, was negative. A second line of chemotherapy with Dactinomycin monotherapy was administered for 4 cycles (dose of 0.5 mg/d from D1 to D5 every 2 weeks). After a fall in hCG levels, a stagnation and then a re-ascension were observed. A new extension work-up using thoraco-abdominal CT-scan and cerebral MRI was reassuring.

After in-depth discussion with both the Belgian and French reference centers for trophoblastic disease, it was decided to propose the use of Avelumab-based immunotherapy as an alternative to polychemotherapy with Etoposide, Methotrexate, Actinomycin D, Cyclophosphamide and Vincristine (EMA-CO). The aim was to reduce long term toxicity and try to preserve natural fertility. Treatment was initiated after the patient’s informed consent had been signed, and after validation by the ethics committee due to the low level of evidence for this therapy in this situation. Unfortunately, no response to immunotherapy was observed.

**Figure 1:**
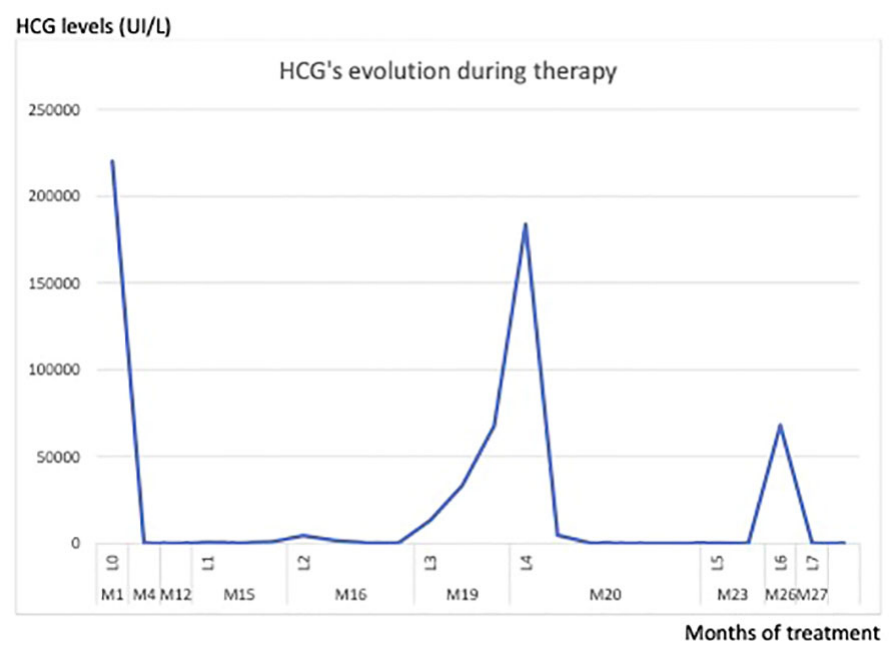
Lines of treatment: L0: curettage, L1: Methotrexate, L2: Dactinomycin, L3: Avelumab, L4: EMACO, L5: Paclitaxel/Cis, L6: Hysterectomy, L7: High dose chemotherapy.

After another negative CT-scan and brain MRI, we had no choice but to start a polychemotherapy. We regretted that the hCG level rose again after the sixth course of EMA-CO. Another line of chemotherapy based on Paclitaxel Platinum - alternating with Etoposide was therefore administered. We rapidly observed an escape from this protocol too. The evolution of hCG over the weeks of treatment is shown in the figure below (**Figure 1**).

Given this resistance to multiple systemic treatments and the absence of distant lesions, we decided to perform an inter-ovarian hysterectomy. The procedure was performed laparoscopically without uterine manipulator, and the specimen was extracted in a single piece. Pre-operative MRI confirmed the presence of a tumoral lesion into the uterus. The operation was performed in March 2023. Pathological analysis of the surgical specimen revealed a choriocarcinoma measuring 5 cm in diameter, located in the anterior wall of the uterine body and infiltrating its entire thickness. Two weeks after the operation, we observed a very rapid progression of the disease, with a hCG level of 67.906 IU/L. A CT-scan revealed multiple lung metastases.

After national and international multicenter consultation, the patient was referred for hematopoietic stem cells auto-transplantation in order to undergo chemotherapy intensification. Intensification treatment consisted of two courses of Ifosfamide-Paclitaxel with normalization of the hCG marker but not of the thoracic CT-scan, followed by three courses of high-dose Carboplatin/Etoposide under stem cells coverage. The regimen was alternated with Neupogen, a recombinant form of hematopoietic growth factor, before her stem cells were harvested. The patient finally went into complete biological and radiological remission after 1 year of intensification, more than 2 years after the initial molar pathology.

## 3 Discussion

Gestational trophoblastic neoplasia is an extremely rare gynecological tumor. Most of them result from the malignant evolution of a benign molar pregnancy. Transformation to GTN occurs in around 15–20% of CHMs (of which 3% develop into choriocarcinoma) and only 0.5 to 5% of PHMs (1–4). However, post-molar GTN is very rare after hCG normalization, occurring in only 0.35% of cases after CHM (5).

The particular complexity of our case already lies in the fact the classic FIGO criteria were not met to make a rapid diagnosis of GTN transformation (6, 7). The first post-MHC hCG test was negative in May 2021. During follow-up, a rise in hCG was noted in January 2022, i.e. after 8 months of negativation. The initial hypothesis was the development of a separate pregnancy labeled “pregnancy of unknown location” but was refuted after diagnostic laparoscopy. The diagnosis of post-molar GTN was retained. Thus, to determine the most appropriate treatment for this disease, we calculated its prognostic score based on the FIGO 2000 classification (**Table 1**).

**Table 1:** FIGO 2000 prognostic classification.

Score	0	1	2	4
**Age**	< 40	≥ 40		
**Antecedent pregnancy**	Hydatidiform mole	Abortion	Term	
**Interval months from index pregnancy**	< 4	4 – 6	7 - 12	≥ 13
**Serum hCG (UI/L)**	< 10^3^	10^3^ – < 10^4^	10^4^ – < 10^5^	≥ 10^5^
**Largest tumor size (including uterus)**		3 – < 5 cm	≥ 5 cm	
**Site of metastases**	Lungs	Spleen, kidney	Gastro-intestinal	Liver, brain
**Number of metastases**	0	1 – 4	5 – 8	> 8
**Previous chemotherapy**	No		Failed monochemotherapy	Failed polychemotherapy

The initial risk score was 4, with disease confined to the uterus (stage I). This was a low-risk score, justifying the initiation of methotrexate-based monochemotherapy as first-line treatment (8). GTN is highly chemosensitive tumour and the remission rate for this risk category is generally close to 100% (9). Treatment had to be stopped during the 3rd cycle, as hCG levels had been rising since the end of the 2nd cycle, indicating resistance to this first-line of monochemotherapy (10, 11).

Another monochemotherapy based on Actinomycin D had been introduced. Based on experience of the French reference center for trophoblastic diseases, it enables most patients who have developed resistance to MTX to catch up, with a complete response rate of 75%, while avoiding the short- (alopecia, asthenia, myelotoxicity, and renal failure) and long-term toxic effects (such as myelodysplasia, leukemia and infertility) of multi-agent therapy with EMA-CO (11).

Unfortunately, after 4 cycles, a further increase in hCG levels was observed. This observation is probably partly explained by the fact that the interval between the initial molar evacuation and the start of methotrexate treatment was greater than 7 months, which is a predictive factor for failure of second-line Dactinomycin therapy (11). The failure of 2 lines of chemotherapy led us to suspect an unrecognized chemoresistant GTN of the PSTT or ETT type, but anatomopathological analysis of some biopsies invalidated this diagnostic hypothesis.

After consultation with the Belgian and French Reference Center for Trophoblastic Diseases, we advised treating our patient with an emerging therapy in this indication. Over the last decade, immunotherapy has already proved its effectiveness in the treatment of melanoma, lung cancer and kidney cancer, and is revolutionizing the treatment of many other cancers (12, 13). GTNs have a particular genomic profile with a high paternal predominance capable of activating an immune response, making them an ideal target for immunotherapy (14, 15). This new therapeutic approach is based on the fact that the programmed death ligand 1 (PDL-1) binding protein is highly expressed in normal placentas and in all GTD subtypes (16–23). In tumors, PD-1 binding to PDL-1 inhibits T-cell activity. The use of PD-1/PDL-1 inhibitors can block this pathway, enabling T cells to destroy tumour cells (19). Given these facts, immune checkpoint inhibitors targeting PD-1 and PDL-1 appear to be effective in the treatment of GTN (12–31). This appears to be a promising way of avoiding combination of chemotherapy and its effects on the quality of life of young patients. Their efficacy has already been demonstrated in multidrug-resistant cases, but their role at an early stage of treatment is emerging (20–31).

The first ICI used for multi-drug resistant GTN was Pembrolizumab, a PD-1 inhibitor, by Ghorani et al. and they showed a complete response (CR) of 75% (3/4 patients). The literature describes only case reports and series concerning the use of immunotherapy in these situations (**Table 2**) (20–29). More recently Braga et al. reported a series of 3 cases of CR using this drug (25, 26). Unfortunately, this molecule is not available in Belgium for this indication. The only prospective trial available at the time is the TROPHIMMUN conducted by the team of You et al. This showed that Avelumab, an anti PDL-1 monoclonal antibody, could cure around 53% of GTN patients resistant to mono-chemotherapy (31). In their study, 15 patients received Avelumab at a dose of 10 mg/kg IV every 2 weeks until hCG normalization, followed by 3 additional consolidation cycles. A complete relapse-free response was observed after 29 months in 8/15 patients. In this cohort, only one patient, like ours, had received Actinomycin D in addition to MTX. Although this treatment has not yet been validated in this indication, given the low level of evidence, we proposed it to our patient in the hope of avoiding combined chemotherapy with its long-term toxic effects and preserving natural fertility (23, 24, 33). In their study, one cured patient was subsequently able to carry a pregnancy to term.

**Table 2:** Some GTN cases treated with immunotherapy reported in the literature.

Authors	Immunotherapy	Prior treatment	Patients	CR after immunotherapy
Huang et al. 2017 (32)	Pembrolizumab	GTN resistant to multiple lines of therapy	1	1
Ghorani et al. 2017 (26)	Pembrolizumab	GTN resistant to multiple lines of therapy	4	3/4
Choi et al. 2019 (27)	Pembrolizumab	GTN resistant to multiple lines of therapy	2	1/2 (partial response for the second patient)
You et al. 2020 (31)	Avelumab	GTN resistant to monochemotherapy	15	8/15
Braga et al. 2023 (25)	Pembrolizumab	GTN resistant to multiple lines of therapy	3	3/3

In view of these encouraging, albeit limited, results, the National Comprehensive Cancer Network has included immunotherapy in its guidelines as an alternative treatment chemotherapy resistant GTN.

Unfortunately, our patient’s hCG level continued to rise, from 33.000 to 115.212 IU/L in 3 weeks, indicating resistance to treatment as defined in this protocol (> 20% increase in hCG level sustained over 3 weeks or plateau with < 10% decrease over 3–4 weeks) (31). The mechanisms of resistance to immunotherapy are not yet well understood, and although its use looks promising in the treatment of GTN, further work is needed to assess which patients would benefit most (20, 23, 26). There is currently only one clinical trial investigating on the use of ICI in the management of GTN. Others are likely to emerge in the coming years, although the rarity of this disease makes the task difficult.

Given the progression of the disease and the fact that the work-up remained unchanged, we had no choice but to start combined EMA-CO chemotherapy according to the standard protocol (**Table 3**) with an expected remission rate of 87% (34–37).

**Table 3:** Schema of EMA-CO Chemotherapy.

Day 1	Etoposide Methotrexate Actinomycin D	100 mg/m^2^ intravenous (IV) 100 mg/m^2^ IV push and 200 mg/m^2^ IV infusion 0,5 mg IV push
Day 2	Etoposide Actinomycin D Folinic acid	100 mg/m^2^ IV 0,5 mg IV push 15 mg intramuscular or orally every 12 hours for 4 doses
Day 8	Vincristine Cyclophosphamide	1 mg/m^2^ IV push 600 mg/m^2^ IV

Once again, we were confronted with resistance to this polychemo-therapy after 6 cycles, despite a good initial response. Guidelines for these patients are not clearly established. Small studies have shown the efficacy of the alternative Paclitaxel-Etoposide and Paclitaxel-Cisplatin (TE-TP) regimen every 2 weeks, with the advantage of introducing new drugs in multi-resistant patients (36). Chemoresistance was observed after 7 cycles.

This resistance to several lines of chemotherapeutic agents nevertheless led us to suspect the presence of a chemoresistant focus, probably in the uterus. A new FDG/PET-scan and thoraco-abdominal CT-scan were performed at this time. This examination revealed for the first time hypermetabolism in the previously identified uterine dome site. In view of these findings and the resistance to 4 lines of chemotherapy and immunotherapy, we had no choice from an oncological point of view but to propose a hysterectomy to the patient, despite her young age and persistent desire for pregnancy.

The option of partial hysterectomy was discussed with the patient, although there are still only a few reported cases in remission who have undergone such an operation and achieved pregnancy with live birth (38, 39). After discussion with the couple, the choice of treatment was total hysterectomy with ovarian preservation. The anatomopathological and immunohistochemical diagnosis was that of a 5 cm diameter choriocarcinoma.

Significant and rapid progression of hCG levels postoperatively, associated with new lung metastases, necessitated the urgent resumption of chemotherapy. There is currently no consensus on the treatment of a condition as rare as this, but several small cohort studies have been carried out on the use of high-dose chemotherapy with hematopoietic stem cell autologous transplantation as a treatment of last resort (40–43). HDC is widely used in the treatment of hematological malignancies and has long been studied in the treatment of breast cancer, germ cell tumors and small cell lung cancer. From 1991 to 2016, only 28 cases of GTN treated with HDC have been reported in the literature. A complete response was observed in 13 of them. In 2018, Frijstein et al. presented the results of the world’s largest series of 32 patients, 13 of whom survived (41%) after a median follow-up time of 55 months (40). The patient received a regimen based on Paclitaxel, Ifosfamide, Carboplatin and Etoposide, as in the majority of cases described in the literature in this indication (43). She received a total of 3 autologous transplants. We are currently awaiting the long-term results of this final line of treatment, but the patient is still in remission with a complete serologic response one year after the end of the treatment.

## 4 Conclusion

This clinical history illustrated an extremely rare case of GTN resistant to multiple chemotherapies and even surgery. This clinical case report illustrates the therapeutic lines currently available for the treatment of this type of gynecological tumour, and discusses emerging immunotherapy in this field, notably with an anti-PDL1 monoclonal antibody called Avelumab. The initial results with Avelumab are encouraging, but require a higher level of evidence. Moreover, we do not know if ICIs should be given alone or associated to chemotherapy. Our case also shows that high-dose chemotherapy with autologous transplantation can be effective in GTN resistant to multiple regimens of chemotherapy.

## Data availability statement

The original contributions presented in the study are included in the article/supplementary material, further inquiries can be directed to the corresponding author/s. 

## Ethics statement

Written informed consent was obtained from the individual(s) for the publication of any potentially identifiable images or data included in this article.

## Author contributions

AE: Conceptualization, Investigation, Visualization, Writing – original draft, Writing – review & editing. LD: Project administration, Resources, Supervision, Validation, Writing – review & editing. CP: Resources, Supervision, Writing – review & editing. JM: Validation, Writing – review & editing. FG: Supervision, Validation, Writing – review & editing.
